# Depression in Rheumatoid Arthritis: A Narrative Review—Diagnostic Challenges, Pathogenic Mechanisms and Effects

**DOI:** 10.3390/medicina58111637

**Published:** 2022-11-13

**Authors:** Cătălina-Elena Ionescu, Claudiu Costinel Popescu, Mihaela Agache, Georgiana Dinache, Cătălin Codreanu

**Affiliations:** 1 Rheumatology Department, “Carol Davila” University of Medicine and Pharmacy, 020021 Bucharest, Romania; 2 “Dr. Ion Stoia” Clinical Center of Rheumatic Diseases, 020983 Bucharest, Romania

**Keywords:** depression, disease activity, quality of life, rheumatoid arthritis

## Abstract

Depression is one of the most frequent comorbidities in rheumatoid arthritis (RA); it takes an important toll on the quality of life of these patients and also leads to a decrease in life expectancy. The current article is a narrative review on depression in RA, with the objective to emphasize and raise awareness on the high prevalence, pathogenic mechanisms, and effects that depression has on RA patients. In RA, the prevalence of depression has been shown to be 2 to 3 times higher than in the general population, with a meta-analysis reporting that 16.8% of RA patients have a major depressive disorder. Future studies are needed to determine the most accurate self-reported depression questionnaires and their ideal threshold for defining depression as compared to diagnostic interview as gold-standard for patients with RA to allow better comparisons across studies. The pathogenesis of depression remains to be fully understood, but recent specialty literature suggests that immune-mediated processes are involved and that there are similarities between the neural networks recruited in inflammation and those implicated in the pathophysiology of depression. Depression in patients with RA is associated with poor long-term outcomes. Multiple studies have shown that depression in RA is associated with increased pain, fatigue, and physical disability. It alters treatment compliance, causes more comorbidities, and leads to higher mortality, partly through increased suicide risk. Depression in RA also increases health service utilization and healthcare costs directly through hospitalization, but also indirectly through loss of work productivity. Assessing depression could be a significant psychomarker of rheumatological outcome in RA.

## 1. Introduction

Rheumatoid arthritis (RA) is the most common form of inflammatory rheumatological disease, affecting 0.5–1% of the population and leading to major socioeconomic consequences [1,2]. It represents a chronic immune-mediated inflammatory process predominantly of the synovial joints, which leads to early progressive irreversible osteo-articular damage, significant functional deficit, secondary osteoarthritis, and even the need for orthopedic surgical interventions (such as total arthroplasty of the hips [3] and knees). It is frequently associated with serious extra-articular systemic manifestations that lead to a 5–10-year reduction in life expectancy [1,2].

One of the factors contributing to the decrease in life expectancy in RA are psychiatric disorders—of which mood disturbances such as depression are the most frequent [4]. Depression is very frequently associated with RA, and it takes a big toll on the patient’s quality of life, making it a very important health problem. Taking into account the current treatment strategies in RA, like “treat-to-target” and “tight control”, the best approach would probably be to also address depression for the best possible outcome of RA patients.

The current article is a narrative review on depression in RA, with the objective to emphasize and raise awareness on the high prevalence, pathogenic mechanisms, and effects that depression has on RA patients. As the literature search revealed, the current review is the only narrative review that incorporates the prevalence and diagnostic difficulties of depression in rheumatoid arthritis with the mechanistic pathways of depression in RA, as well as the effects it has on this category of patients, with the objective to grasp this comorbidity from all points of view.

## 2. Materials and Methods

To better understand the problem, the article first focuses on the prevalence of depression in RA and the difficulties encountered when trying to diagnose this mood disturbance in this category of patients. Afterwards, the article highlights the pathogenesis of depression and especially the common mechanistic pathways that depression shares with RA. The last chapter is dedicated to the effects that depression has on RA patients, the way it influences disease activity, and the notions that it alters pain perception and correlates with higher disability.

**Figure 1:**
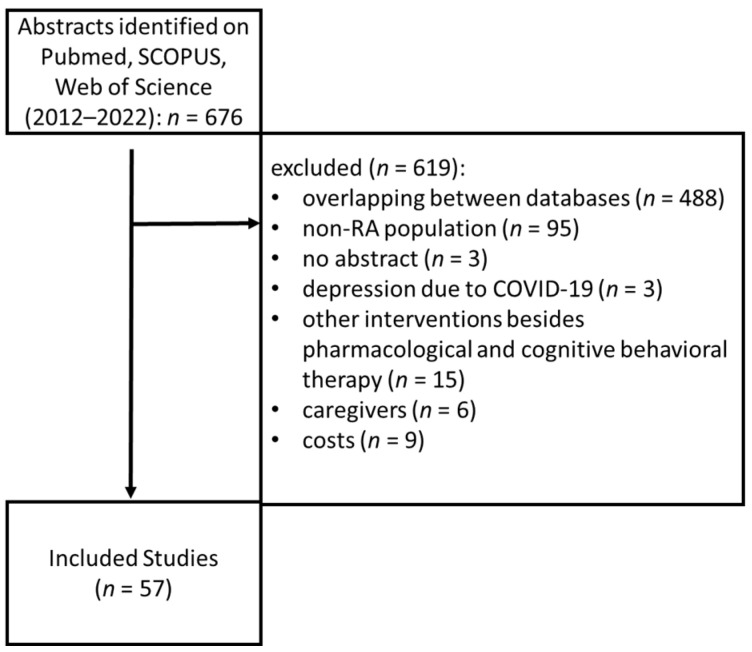
Study selection for inclusion.

Pubmed, SCOPUS and Web of Science Core Collection were searched with the terms: “rheumatoid” and “depression”, restricted to the titles, between 2012 and 2022, which identified 676 articles. Inclusion criteria were as follows: prevalence studies of depression in RA, reviews or meta-analyses relating to disease pathology, studies about therapies for depression in RA, studies on the impact of depression in quality of life and outcomes of patients with RA. Exclusion criteria were: studies that had no available abstract, studies that included patients with other rheumatological diseases than RA, studies that included other interventions besides pharmacological and psychological interventions, studies that included reactive depression to COVID-19, and studies that included healthcare costs. Additionally, the bibliographies of all the articles included were reviewed. After screening and applying the inclusion and exclusion criteria, 57 articles remained in the study (Figure 1).

## 3. Results

### 3.1. Prevalence of Depression in RA

In the general population, depression has a prevalence of 6% [5]. However, in RA, the prevalence of depression has been shown to be 2–3 times higher than in the general population, with a meta-analysis reporting that 16.8% of RA patients have a major depressive disorder [6]. Furthermore, a cross-sectional study showed that depression was more prevalent in RA than in patients with diabetes, cancer, and Parkinson’s disease [7]. This finding may be related to several factors that make patients with RA more vulnerable to developing depression, such as chronic pain and physical disability leading to inability to work, as well as potential side-effects from disease modifying anti-rheumatic drugs (DMARDs) and glucocorticoids [7].

On the other hand, multiple studies have shown a high variability between countries in the prevalence of depression in RA patients, ranging from 2% in Morocco to approximately 33% in the USA [6]. This high variability in the clinical studies of RA is most likely caused by the differences of study quality, the variations in the definitions of depression, and the methods used for measuring depressive symptoms [8]. Socio-cultural differences are also a very important factor in the variation of prevalence. RA itself poses specific challenges to depression diagnosis, and its pathogenic mechanism is actively involved in the appearance of this psychiatric disorder, as is explored in the following sections.

### 3.2. Challenges in Defining Depression in RA Patients

In clinical practice, differentiating between patients with depressive disorders and the adequate reaction has proven to be challenging in patients with chronic debilitating diseases. Furthermore, patients with RA also have constitutional manifestations like fatigue, weight loss, insomnia, and lack of appetite, which overlap with the symptoms of depression. Moreover, our literature search revealed no studies attempting to differentiate the spectrum of depression in RA patients, namely to discriminate between depressive symptoms, depressive syndromes, and depression as a unique nosological entity (unipolar mood disorder).

The gold standard of detecting depression is the psychiatric interview and diagnosis according to Diagnostic and Statistical Manual (DSM) or International Classification of Diseases (ICD) criteria [9], which are time consuming, expensive, and not ideal for assessing patients in a busy medical environment. However, in clinical research studies on the prevalence of depression [10,11], this is more often defined by using self-reported screening questionnaires, which are quicker, easier to complete, and cheaper. Compared to the gold-standard, self-reported questionnaires may result in the overestimation of prevalence, as screening tools tend to prioritize sensitivity over specificity due to low predefined thresholds for diagnosis [9].

A meta-analysis from 2013 showed that one of the main problems with using self-reported patient questionnaires is the variability in defining the term ‘depression’, with more than 40 encountered definitions [10,11]. This emphasizes the need for screening tools to be validated against a clinical interview as the gold standard in order to assess depressive symptoms [12]. The present article was not able to investigate whether other diseases or specific drugs were used as exclusion criteria for the diagnosis of depression based on self-reported screening questionnaires.

A meta-analysis on depression definitions in RA patients showed that some of the screening tools used to detect depression are Patient Health Questionnaire-9 (PHQ-9), Inventory to Diagnose Depression (IDD), Hospital Anxiety and Depression Scale (HADS), Center for Epidemiological Studies Depression Scale (CESD), Beck Depression Inventory (BDI), and the Geriatric Depression Scale (GDS), the most frequently used being the HADS and the CESD. The definition of depression based on these questionnaires included possible or probable positive diagnosis by using either one or multiple thresholds [13]. For example, the CESD, one of the most commonly used questionnaires, was used with 9 different cut-off points. Additionally, when using the HADS measure, the range of estimates for depression was between 14.8% and 48% due to the use of variable cut-off scores [14].

Moreover, not only is there variability between studies using the same self-reported questionnaire, but there is also a large overlap between systemic RA symptoms indicating disease activity and depression symptoms [14]. Therefore, no single tool should be favored for depression assessment in RA patients [15], and all screening tools need clinical diagnostic confirmation [16].

Future studies are needed to determine the most accurate self-reported depression questionnaires and their ideal threshold for defining depression as compared to the gold-standard for patients with RA in order to allow better comparisons across studies.

### 3.3. Mechanism of Depression in RA

The co-occurrence of immune-mediated inflammatory diseases and depression has been recognized for a long time [17]. Studies show that immune-mediated inflammation affects both neuroendocrine activity and neuroplasticity [18].

#### 3.3.1. Inflammatory Mechanisms

RA is a well-known immune-mediated inflammatory disease characterized by high levels of major proinflammatory cytokines, such as interleukin-1β (IL-1β), tumor necrosis factor-α (TNFα), and IL6, suggesting dysregulations between the innate and adaptive (auto) immune system pathophysiology [19].

It has been shown that systemic inflammation may contribute to the development of depressive symptoms during different disorders of chronic inflammation, such as diabetes mellitus and chronic obstructive pulmonary disease [14]. The pathogenesis of depression remains to be fully understood, but recent literature suggests that immune-mediated processes are involved [20].

This has been supported by meta-analyses showing high levels of the same proinflammatory cytokines like RA (IL6, IL1β, and TNFα) in the peripheral blood of patients with depression compared with controls [21]. The role of these cytokines in depression is supported by the similarities between the symptoms of cytokine-induced sickness behavior and depression such as behavioral inhibition, anorexia, weight loss, anhedonia, psychosomatic symptoms, anxiety, and neurocognitive symptoms [22].

Inflammatory cytokines may enter the central nervous system (CNS) through a humoral or neural route. The humoral route passes through the blood-brain barrier, which becomes permeable due to circulating mediators and can then transport molecules into the CNS (e.g., TNFα) [23]. The neural route of communication is best described by the inflammatory reflex model that involves the vagus nerve. It has been proposed that peripheral immune status is transmitted through the vagus nerve by ascending afferent information to the CNS, and the peripheral immune response is modulated by descending efferent information through the vagus nerve [24].

#### 3.3.2. Modulatory Effects of Cytokines in Depression

One important cytokine involved in depression, which is upregulated in RA, is IL1β. IL1β is a key mediator in stress responses, and it is found in abundance in the hippocampus, hypothalamus, and cerebral cortex. The hippocampus is an important structure in the pathophysiology of depression through the hypothalamic-pituitary-adrenal (HPA) axis. This cytokine has been shown to be upregulated in blood and cerebrospinal fluid in patients with depression and life stressors. It has also been shown to correlate with the severity and duration of depressive symptoms [25]. Goshen et al. showed that IL1β is necessary and sufficient to induce depression in mice through the HPA axis [26].

IL-6 is another important cytokine involved in inflammation and depression. A recent study from 2009 showed that IL-6 is increased in the subgenual anterior cingulate cortex of healthy male volunteers who develop mood deterioration within 3 h of seeing an emotional facial expression. This region is similarly involved in primary depression, suggesting that this is a common mechanistic pathway with an immune-mediated inflammatory trigger [27].

Another molecule important in linking RA and depression is type 1 interferon (IFN). IFN is usually involved in the viral inflammatory response. However, it has also been linked to neurotransmitters, such as glutamate, in the basal ganglia and implicated in the pathogenesis of mood changes [17].

#### 3.3.3. Inflammation in Pain and Fatigue

Cytokines, such as IL6, TNFα, and IL1β, are not only involved in the pathogenic mechanism of depression, but also of pain and fatigue. Thus, pain and fatigue as primary manifestations of RA could indirectly lead to depressive pathology. Pain and fatigue are often not addressed completely by DMARDs and are associated with higher levels of depression [28]. However, anti-IL6 DMARDs do show an improvement in pain and fatigue with lower levels of depression [28]. Controlling symptoms such as pain and fatigue by inhibiting IL-6 may prove promising for decreasing depression levels in patients with RA. RA activity has been shown to correlate with high plasma levels of C-reactive protein (CRP), as well as depression severity in this subgroup of patients [29]. CRP levels also correlate with treatment-resistant depression, which could explain why patients with RA are more susceptible and less responsive to antidepressant therapy [30].

#### 3.3.4. Effect of Inflammation on Neurotransmission

It is a well-known fact that low serotonin levels are correlated with depressive symptoms [31]. Inflammation has been shown to decrease serotonin availability within the CNS or the brainstem through either lack of its precursor, tryptophan, or increased serotonin transporter activity. In inflammatory diseases, TNFα expression is increased, leading to high levels of the serotonin transporter, which lowers serotonin levels in the brainstem. As mentioned above, TNFα levels are increased in RA patients [17].

Moreover, in immune-mediated inflammation, indoleamine 2,3-dioxygenase (IDO) is upregulated and is an enzyme that leads to the breakdown of tryptophan. The breakdown products of tryptophan can have both neuroprotective and neurotoxic effects. The ratio of breakdown products is skewed in inflammatory conditions towards neurotoxic effects. This pattern correlates with mood disorders [17]. In inflammation, the IDO pathway is also upregulated in mature dendritic cells, leading to reverse signaling to regulatory T-cells contributing to autoimmunity [32].

Another important molecule for neurotransmission and neuroprotection is brain-derived neurotrophic factor (BDNF). Inflammation has been shown to decrease its levels and its effects on neurogenesis. RA animal models showed that these changes are associated with depression [33]. Furthermore, in rat models, anti-TNF medication DMARDs like etanercept have been shown to increase hippocampal BDNF [34].

#### 3.3.5. Structural Changes in RA Patients with Depression

Structural and functional magnetic resonance imaging (MRI) studies have shown how peripheral inflammation, a hallmark of RA, affects the structure and connectivity of the brain. Schrepf and colleagues found altered patterns of brain connectivity in RA patients with higher levels of disease activity, as well as evidence of inflammation-associated subnetwork reorganization [35].

Other studies looking at depression using whole-brain analysis showed that CRP and inflammatory cytokine levels were associated with decreased connectivity within the reward-related brain regions [36]. Moreover, depressive symptoms, such as low mood, fatigue, and anxiety have been shown to decrease whole brain network efficiency due to IFN-α effects on brain connectivity [37]. However, IFN-α induced fatigue has been shown to behave differently to inflammation on other MRI studies. Basal ganglia may be more sensitive to IFN-α and may be responsible for fatigue in this context. Regardless of the mechanism for fatigue in patients with RA, it has been shown that fatigue and pain can produce and aggravate depression. This could be through shared pathophysiology or through their association with disability and lower QoL [17].

Other neuroimaging human studies support an effect of systemic inflammation on areas that are central to affective control and regulation, such as the subgenual anterior cingulate cortex (sACC), leading to mood changes. Furthermore, it has been shown that peripheral inflammation leads to changes in substantia nigra function and the generation of sickness symptoms, such as psychomotor slowing [38].

Further imaging techniques, such as magnetic resonance spectroscopy, have shown that IFN-α increases glutamate levels in the dorsal cingulate in patients with high inflammation and mood disorders. This suggests that proinflammatory cytokines are responsible for the depressive symptoms found in patients with RA [39].

In summary, this data suggest that similarities exist between the neural networks recruited in inflammation and those implicated in the pathophysiology of depression. RA has been clearly associated with a high prevalence of depression, but recent epidemiological studies imply a bidirectional relationship between these two entities, suggesting patients with depression are also at high risk of developing RA [40]. Therefore, the fact that depression and RA might share a pathogenetic pathway is plausible. Besides sharing common biological pathogenic pathways, the appearance of depression in RA patients does not create a passive association, but it influences the clinical characteristics and the secondary outcomes of RA, as shown in the following sections.

### 3.4. Effects of Depression in RA Patients

Depression in patients with RA is associated with poor long-term outcomes. Multiple studies have shown that depression in RA is associated with increased pain, fatigue, and physical disability. This alters treatment compliance, causes more comorbidities, and leads to higher mortality, partly through increased suicide risk. Depression in RA increases health service utilization and healthcare costs directly through hospitalization, but also indirectly through loss of work productivity. It also considerably decreases quality of life (QoL) for RA patients, a fact that is reflected in all domains of QoL questionnaires [41]. Additionally, psychological distress may impact health outcomes by influencing other health behaviors alongside medication adherence, such as smoking and physical activity. Reduced levels of physical activity can result in deconditioning, loss of natural endorphins, and increased pain [13].

#### 3.4.1. Depression and Altered Pain Perception

Mood disorders, like depression and anxiety, have been incriminated as potential factors for relapse periods in RA patients [42], and they significantly alter the way patients perceive their current health. Patients’ perception of their overall health (global health) is independently and negatively associated with depression. Patients with RA who perceive their health to be poor or who see themselves as disabled, be it related or not to their RA diagnosis, tend to experience high levels of depression [43].

Depression modifies the patient’s perception of their current health status and lowers thresholds of pain, leading to higher disease activity scores and complicating the management of the disease [44]. Despite the use of DMARDs to control disease activity, patients with underlying mood disorders may have persistent pain. Treating these mood disorders might lead to higher pain thresholds and improve the patients’ quality of life.

#### 3.4.2. Depression Correlates with Higher Disease Activity

Multiple studies have shown there is a clear correlation between the severity of depression and disease activity [45]. High depression scores are associated with RA disease activity. However, causality or direction of effect cannot be established from observational studies [46]. A bidirectional relationship between depression and disease activity is likely, as studies in early RA report that higher joint counts at baseline are associated with increased depression at 6 months. Some studies show that depression leads to higher disease activity through association with the subjective components of the disease activity score (DAS28), such as tender joint count or patient global assessment [47]. Reports also found a correlation between baseline depression in patients with RA and higher subjective DAS28 components, such as the global assessments, joint pain, and tender joint counts at 3 or 6 months.

Objective measures, such as swollen joint counts [48] and inflammatory markers of disease activity, like the CRP, have been variably associated with baseline depression in different studies [44]. Some studies support the idea that there is a close relationship between inflammation and depression, as CRP levels were correlated with depression scores at subsequent follow-up time-points. Conversely, higher CRP levels at baseline have also been shown to distinguish between RA patients who would go on to develop depression [46].

#### 3.4.3. Depression as a Negative Predictor of Remission in RA

Depression has been found to be a strong negative predictor of remission in observational study [49], at 3–6 months post-DMARDs treatment of RA patients [47]. Furthermore, randomized controlled trials of tocilizumab and csDMARDs showed that patients with comorbid depression were 40% less likely to achieve remission at any time-point. Nevertheless both antidepressant therapy and inflammation control (for example using conventional synthetic drugs [50] or tofacitinib [51] and rituximab [52]) were reported to lead to improvement in RA activity and treatment response [53,54]. On the other hand, psychological interventions for RA patients, such as behavioral therapies, have been reported to have a minor effect for pain, fatigue, and patient global assessment [48], which are significantly influenced by comorbid depression [55]. Unfortunately, there are few clinical trials that evaluate interventions in RA patients with depression, despite this positive evidence [56].

In conclusion, baseline depression in RA has been shown to decrease disease remission rates, mostly through subjective measures of disease activity. Promoting psychological wellbeing early in the course of disease may be a cost-efficient measure for preventing higher levels of disease activity later on, leading to less aggressive treatment strategies [52].

#### 3.4.4. Depression and Disability

Patients with depression also tend to have poor functional status, partly because of poor radiological outcomes in the presence of depression [57]. On the other hand, any improvement in depressive symptoms may lead to improvement in the functional status of the patient [44]. A Dutch study revealed that fluctuations in the level of depression were associated with fluctuating levels of functional status of RA patients [53].

Indeed, studies showed that the health assessment questionnaire (HAQ) score, a measure of overall patient wellbeing, was the most important predictor of psychological stress in early RA. Other demographic and socio-economical aspects, such as occupation and family income, were associated with depression in RA.

Fragoulis et al. identified a number of factors associated with HADS depression scores at all timepoints as follows: baseline depression scores, unemployment, being single, younger age, HAQ score and increased disease activity measures. Multiple linear regression analysis indicated that current HAQ scores and baseline depression scores were the strongest predictors of depression scores.

The association between HAQ and depression is consistent with findings from another study using data from the Scottish Early RA inception (SERA) cohort. The SERA inception cohort, from Scotland, included patients with new-onset RA, according to the ACR-EULAR 2010 criteria, recruited prospectively, presenting to secondary-care rheumatology centers between 2011–2015. This study reported that functional disability at 1-year after RA diagnosis was predicted by depression among other factors. Similarly, another study examining predictors of depression in a multi-ethnic RA cohort showed that higher HAQ scores were associated with depression [46].

#### 3.4.5. Depression Increases Healthcare Resource Utilization

Depression in RA can also increase healthcare resource utilization, which can lead to high health care expenditures for both insurance payers, patients, and families. The burden of depression has high socioeconomic costs including workplace absenteeism and presenteeism, and workplace productivity is associated with functional disability and depressive symptoms [12]. Moreover, the presence of depression along with any chronic physical condition more than doubles the likelihood of work absenteeism as compared to the presence of any chronic physical condition without depression [58].

## 4. Discussion

Depression is highly prevalent in RA, and the toll it takes on the quality of life of patients suggests that in routine clinical practice and corresponding follow-up procedures, patients should be screened for the presence of depressive symptoms [5]. Diagnosing and treating depression in this category of patients should be part of standard patient care, but that implies using validated screening questionnaires against the gold standard for detecting depression. A full diagnostic assessment should be made after positive screening results [59] in order to confirm the diagnosis and to minimize the risk of attributing depression to RA in the presence of concomitant etiological disease (such as iatrogenic effects or other psychiatric and non-psychiatric disorders associated with depression).

The correlation between rheumatological outcome and depression is strong, without consideration of the direction of causality, so mental health should be addressed as part of a regular procedure in rheumatological randomized controlled trials and cohorts. Assessing depression could be a significant psychomarker of rheumatological outcome. Depression should be assessed before and after starting treatment and also at different time-points during treatment, because there is compelling evidence that depression is highly treatable in patients with co-occurring physical diseases. Disease activity, as assessed by the DAS28 score, is used to make treatment decisions, and the fact that disease activity is influenced by depression scores means that depression scores influence treatment decisions. Correctly detecting and managing depression may be necessary for the effective management of RA [60].

There is a lack of interventional studies in this field. Extensive research should be undertaken in order to evaluate whether treating depression does lead to improved rheumatological outcomes. Further investigation into what type of therapy is the most effective in treating comorbid depression in RA is needed.

There is compelling evidence that cognitive behavioral therapy and mindfulness for depression in RA patients leads to better outcomes. Nursing interventions may be important adjunctive therapies to the medical management of RA patients. Secondly, most studies showed that depression in RA is drug resistant and does not seem to respond well to classical therapy. In line with the treat-to-target and the tight control strategy for disease activity, it has become clearer than ever that depression in RA patients must be addressed.

This review has several study limitations that can influence the conclusions, namely the fact that the literature is vast and highly unlikely to be completely summarized in one review, as well as the possible bias originating from the author’s clinical experience.

## 5. Conclusions

Depression is significantly prevalent among RA patients and is often masked by RA-associated pain and systemic manifestations. The difficulty and variability of diagnosing depression is also caused by the fact that self-reported depression questionnaires have not been accurately compared to the interview gold-standard. Well-designed cross-sectional epidemiological studies are needed to understand the type of depressive disorder (symptoms, syndrome or unipolar mood disorder), the prevalence of depression in patients with RA, and their impact on disease activity.

The two entities share a bidirectional pathogenic relationship that is responsible for the multiple effects of this association, in terms of altered pain perception, higher RA disease activity, lower chance of RA remission, and increased disability and healthcare resource utilization. Assessing depression could be a significant psychomarker of rheumatological outcome in RA.

Further investigation into what type of therapy is the most effective in treating comorbid depression in RA is needed. Prospective studies addressing the best type of therapy for depression in patients with RA would be needed to further increase our knowledge and practical approach to this vital issue.
